# Role of sintered temperature and sintering time on spectral translucence of nano-crystal monolithic zirconia

**DOI:** 10.4317/jced.55497

**Published:** 2019-02-01

**Authors:** Surawut Attachoo, Niwut Juntavee

**Affiliations:** 1 Division of Biomaterials and Prosthodontics Research, Faculty of Dentistry, Khon Kaen University, Khon Kaen, Thailand; 2 Department of Prosthodontics, Faculty of Dentistry, Khon Kaen University, Khon Kaen, Thailand

## Abstract

**Background:**

Sintering process is accountable for aesthetic appearance of zirconia restoration. This study appraised the effect of different sintering procedure via sintered temperatures and sintering times on spectral translucence of monolithic zirconia.

**Material and Methods:**

One hundred and thirty five monolithic zirconia specimens (width, length, thickness = 10, 20, 1.5 mm) were prepared from yttrium-stabilized tetragonal zirconia polycrystalline (Y-TZP, Ceramill®) and unintentionally divided into nine groups to be sintered at different temperatures [decreasing- (SD, 1350°C), regular- (SR, 1450°C), and increasing- (SI, 1550°C) sintering temperature] and different sintering times [shortening- (HS, 60 min), regular- (HR, 120 min), and prolong- (HP, 180 min) sintering time]. Spectral translucence was determined by using spectrophotometer and calculated for translucency parameter (TP). The surface topography and grain size were evaluated by using a scanning electron microscope (SEM). Crystalline structures of monoclinic (m) and tetragonal (t) phases were determined by using the X-ray diffraction (XRD). An analysis of variance (ANOVA) was used to determine for significant differences of translucence upon different sintering processes (α=0.05).

**Results:**

The mean, standard deviation of TP were 3.22±0.12 for SRHP, 3.14±0.18 for SIHS, 3.04±0.17 for SRHR, 2.94±0.18 for SRHS, 2.93±0.17 for SIHR, 2.67±0.15 for SIHP, 1.91±0.17 for SDHP, 1.34±0.21 for SDHR and 0.10±0.01 for SDHS. Spectral translucence was significantly affected by altering sintering temperatures and holding times (*p*<0.05). Enlargement of grain size and increasing t→m phase metamorphosis related with upraising sintered temperatures and extending sintered holding times were signified.

**Conclusions:**

Altering sintering parameters affected spectral translucence of zirconia. Upraising sintered temperature to SR and prolonging sintering time to HP were advocated to enhance spectral translucence of nano-crystal monolithic zirconia, and advised to accomplished aesthetic appearance of restoration in clinical practice.

** Key words:**CAD-CAM, sintering process, translucency, zirconia.

## Introduction

The esthetic demands in dentistry have been intensifying the evolution of modern dental ceramics in the field of restorative dentistry. Ceramic restorations have been rising as a restoration of choice replacing ceramic veneered metal restorations, owing principally to their honorable appearance, corrosion resistance, and biological compatibility (1). Nevertheless, the brittleness of ceramic has restricted their applications in extensive restoration. Some ceramics such as glass-infused aluminous oxide-, leucite reinforced-, and glass-containing lithium disilicate ceramics have inevitably been utilized in crowns or 3-units bridge as they do not retain the tolerable strength for extensive restorations. Lately, zirconia ceramics have become progressively desirable due to their authentic strength in comprehensive reconstruction (2). Generally, zirconia comprises of changeable crystalline structure and occurs in three phases together with monoclinic (m), tetragonal (t), and cubic (c). The monoclinic crystal system occurs between normal room temperature and 1173°C. Beyond this, it converts to a dense tetragonal phase and is structurally stable until proceeding through 2370°C, during which the cubic phase occurs until the melting temperature of 2690°C is reached (3). The t- → m-phase transformation can be seen upon solidification of the natural zirconia, which is 3–5% of volumetric enlargement. Nevertheless, the solidification of zirconia may be regulated to crystalize it in t-phase by mixing yttrium-oxide (Y2O3) particles for approximately 3.5–8.7%, resulting in a modern yttrium partially stabilized tetragonal zirconia polycrystal (Y-TZP) that is competent enough to prevent generation of cracks as a result of a transformation toughening procedure, which enhances the resistance to fracture from the masticatory system in advanced reconstruction.

The zirconia prostheses are usually constructed using digitally controlled technology by milling either a partial sintered blank that needs additional sintering to complete the sintering process or a fully-sintered blank that does not require an additional sintering procedure but causes fast wearing of the milling machine (4). Though milling partially sintering blank is suitable for machining, they may compromise the accuracy of restoration due to the shrinkage of zirconia upon sintering (5). The zirconia exhibits a white opaque color scheme with minimal translucency and requires veneering with glassy ceramic to derive a natural appearance (6). However, the dental glass ceramic generally possesses inferior tensile strength capable of withstanding masticatory stress. Thus, the delamination or splitting of glass ceramic from zirconia substructure happens regularly, becoming an annoyance situation in dental practice (7). The introduction of monolithic zirconia was aimed at elimination of the ceramic chipping problem; however, the possibility of achieving aesthetic appearance that can replicate the color characteristic of the natural tooth is still restricted. Currently, monolithic zirconia with high translucency was introduced, and gained awareness to clinician attributable to its unique color characteristics and durable strength through a nano-sized crystal structure (< 500 nm) that possibly diminished the scattering phenomenon and probably contributed a natural tooth appearance (8).

The light characteristic of monolithic zirconia needs to be simulated to that of the tooth structure for the restoration to derive a natural appearance. Nonetheless, during the construction process, numerous factors may influence the optical behavior of the Y-TZP, for instance the size of the zirconia particle, heating process, and sintered procedure. However, the principal determinants affecting the densification and crystallization of zirconia are the sintered temperature and sintering time (9,10). The optical characteristics predominantly relate to the spectral reflectance that is a result of the scattering effect of light at the surface and are measured by the Commission Internationale de l’Eclairage (CIE) color space as a color quantification by physiologic human perception is relatively subjective (11,12). Optical characteristics that are distinguished based on translucence, opalescence, and contrast are essential determinants for shade selection in dentistry. Translucence is considered a capability of light transmitting through a diffusely material and is comparatively related to the wavelength of light scattering phenomenon as a result of crystal arrangement, dispersion of porosities, and density of material (10,13,14). The material would become opaque if the majority of light passing through the material was vigorously reflected and scattered. Conversely, it would become translucent if a majority of the light strongly passed through the bulk of material with diminutive scattering and tiny reflectance (13). Since the zirconia comprised numerous crystalline forms that possess different index of refraction and non-homogeneous crystalline configuration, it generally displays fiercely scattered effect and diffused reflection and causes an opaque situation. The translucent property of material is usually measured in terms of the translucency parameter (TP) (15,16). The TP designates the difference in color of a material upon the black and white background at a constant thickness and correlate relatively with the visual determination of translucency. The translucency parameter that equals to zero stipulates an absolutely opacity whereas the greater number of translucency parameter stipulates higher material translucency.

The improvement of the optical properties of zirconia restoration is possible for dentists through the zirconia sintering procedure (14). The optical characteristics of zirconia have been described to enhance translucency by adjusting the sintering process to directly influence the crystalline structure of zirconia (17-19). It was reported that an alteration in the temperature and time for sintering process can influence the crystal size, crystalline structure as well as the optical characteristics of zirconia (20-22). Upon enlargement of grain size, zirconia may be instinctively changeable from its t-phase to m-phase, which may affect its optical appearance (23-25). This is of great important in dentistry specifically in relation to the short duration of sintering process as described by the manufacturers. Furthermore, the impact of the sintering agenda on the spectral characteristics of the nano-crystal Y-TZP is yet contentious. The present investigation determined even if the variation of time and temperature for sintering affected the translucency of the nano-crystal zirconia. The null hypothesis was that the alteration of sintered temperature and sintering time might not influence the translucence of nano-crystal monolithic zirconia.

## Material and Methods

-Zirconia samples preparation

The bar-shaped Y-TZP samples [135 pieces at 12 mm width (W), 25 mm length (L), 1.8 mm thickness (T)] were produced from pre-sintered blanks (Ceramill® Zolid classic, Amann Girrbach, Koblach, Austria) using sectioning apparatus (Isomet®, Beuhler, Lake Buff, IL, USA). The zirconia bar samples were serially polished up to 2,400 grit of carborundum and one micron (μm) diamond suspended liquid in a grinding apparatus (Ecomet®, Beuhler, Lake Bluff, IL, USA) to derive the final dimension. The samples were cleansed in distilled water, desiccated for 60 minutes (min), and unintentionally divided to nine groups (15 samples each) based on the sintering procedure at three sintered temperatures including decreasing- (SD, 1350°C), regular- (SR, 1450°C), and increasing- (SI, 1550°C) sintered temperature, together with three durations of sintering times, including shortening- (HS, 60 min), regular- (HR, 120 min), and prolonged- (HS, 180 min) sintering time, as shown in  Table 1. All the samples were sintered with furnace (inFire®, Sirona, Bensheim, Germany) at the rate of 8°C/min heating and 5°C/min cooling. The final dimension of each bar sample (W x L x T = 10 x 20 x 1.5 mm) was derived as a result of the sintering shrinkage of roughly 20% of the volume.

**Table 1:**
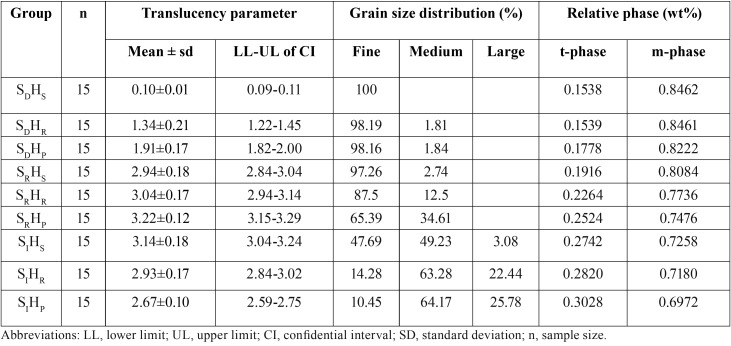
Mean, standard deviation (sd), 95% confidence interval (CI) of translucency parameter (TP), grain size distribution (%), and relative phase content (wt%) of zirconia, sintered at decreasing- (SD), regular- (SR), and increasing- (SI) sintered temperature, with shortening- (HS), regular- (HR), and prolonged- (HP) sintering time.

-Determination of translucency 

Translucency was determined by measuring CIE L*a*b* orders for all samples against a white background (CIE a* = 0.1, L* = 96.7, b* = 0.2) along with those in black background (CIE a* = 0.4, L* = 10.4, b* = 0.6) with color spectrometer (ColorQuest®, Hunter, Reston, VA, USA). The D65 illuminant with a standard wavelength between 300–780 nm at 6504 K of coloring temperature was used at a 10 degrees observer angle and calculated for translucency parameter (TP), according to equation 1. A sample was measured with 4 mm in ϕ of aperture size at the mid-portion area using a positioning device.

TP= [(LB-LW )2+(aB- aW )2+ (bB- bW )2 ]1⁄2....Equation 1

Where, L: brightness, a: redness-greenness, b: yellowness-blueness, B: measured upon black background, and W: measured upon white background.

-Microscopic examination for zirconia

The surface of the sample was ultrasonically cleaned in distilled water, dried in the desiccator, and sputter-coated with gold-palladium for 3 minutes at 10 mA current and a 130 Torr of vacuum in the coating machine (K 500X, Emitech, Asford, UK) prior to the evaluation for a microscopic structure using a scanning electron microscope (SEM, Hitachi, Tokyo, Japan) under 30,000x magnification.

-Analysis of microstructure of crystal

The crystal structure of zirconia was evaluated with the X-ray diffractometer (XRD, PANalytical, Empyrean, Almelo, Netherland). The copper k-alpha (Cu Kα) radiation, with 0.15418 nm wavelength (λ), was used to scan for 2 seconds interval, from 20–40o of the Bragg angle (2θ) at 0.02o step angle. The XRD micrograph was compared with those in the standard database and calculated for a distance of khl-Miller indices (d), as given in Equation 2.

λ = d2 sin θ....Equation 2

The peaks’ intensities for m- and t-phase (Im & It) were computed using the software (X’Pert Plus, Philips, Almelo, Netherland) for m-phase fraction (Xm), as given in Equation 3, corrected with composition-dependent factor (C = 1.32) for un-linearity, as in Equations 4, and also calculated for mass fraction of t-phase (Xt), as in Equation 5 (26).

**Figure 1:**
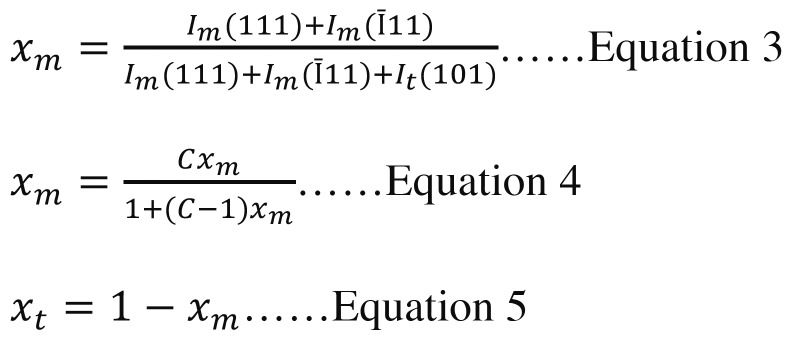
Equations 3,4,5.

-Statistic determination: (Fig. 1).

The data was inspected for significant differences in translucency upon different sintering temperatures and sintered-holding times using a statistical package for social sciences Version 20 (IBM, Armonk, NY, USA) and further evaluated to determine the significant effect upon each factors with Post-hoc Tukey’s multiple comparison at α=0.05.

## Results

**Figure 2:**
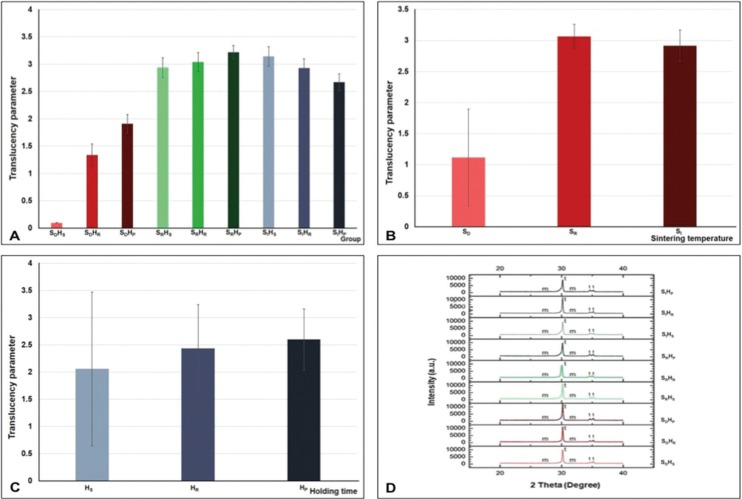
Translucency parameter (TP) of Ceramill Zolid® monolithic zirconia, sintered at decreasing- (SD), regular- (SR), and increasing- (SI) sintered temperature, together with shortening- (HS), regular- (HR), and prolonged- (HP) sintering time (A), indicated the effect of sintering procedure via different sintered temperature (B), and sintering time (C) and related with the X-Ray diffraction pattern of monolithic zirconia (D).

The TP for each group was presented in terms of mean, standard deviation (sd), and 95% confidence interval (CI), as given in Table 1. The highest TP value was demonstrated in the group SRHP (3.22±0.12), followed by SIHS (3.14±0.18), SRHR (3.04±0.17), SRHS (2.94±0.18), SIHR (2.93±0.17), SIHP (2.67±0.15), SDHP (1.91±0.17), SDHR (1.34±0.21), and SDHS (0.10±0.01), accordingly, as indicated in  Table 1 and Figure 2(A).

ANOVA exhibited statistically significant differences in TP due to altering sintering-temperatures and times of Y-TZP sintering procedure (*p*<0.05), as indicated in  Table 2. The difference between the groups of TP demonstrated that sintered zirconia at regular sintered-temperature caused significantly better translucency than at decreasing- and increasing-sintered temperature whereas sintered zirconia at a decreasing sintered temperature caused significantly reducing translucency than at increasing sintered temperature, as indicated in  Table 3 (A) and Figure 2 (B) (*p*<0.05). The difference in the sintering time in the different groups of translucency parameter indicated that the prolonged sintering time for zirconia caused significantly better translucency than at shortening- and regular-sintering time (*P*<0.05). Moreover, sintered zirconia at a decreasing sintered-holding time contributed to significantly reducing translucency than at a regular sintering time, as revealed in  Table 3 (B) and Figure 2 (C) (*p*<0.05).

**Table 2:**
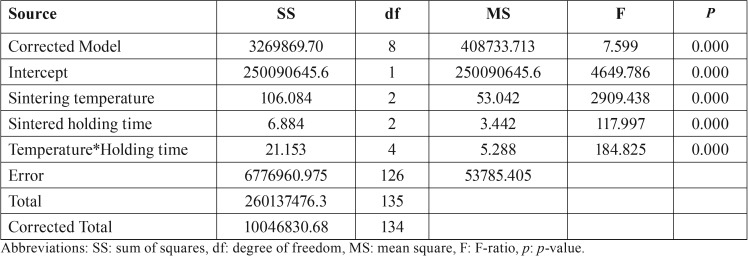
An analysis of variance (ANOVA) of translucency parameter (TP) of monolithic zirconia, sintered at decreasing- (SD), regular- (SR), and increasing- (SI) sintered temperature, with shortening- (HS), regular- (HR), and prolonged- (HP) sintering time, indicated the effect of sintered temperature and sintering time on spectral translucence.

**Table 3:**
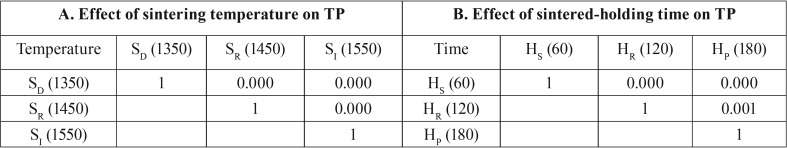
Post hoc Turkey multiple comparisons of translucency parameter (TP) of monolithic zirconia, sintered at decreasing- (SD), regular- (SR), and increasing- (SI) sintering temperature, with shortening- (HS), regular- (HR), and prolonged- (HP) sintering time, indicated the effect of (A) sintered temperature and (B) sintering time on spectral translucence.

The crystalline phase evaluation of the samples with XRD indicated that the spectral peak positions for specimen corresponded with the t- and m- phases for ZrO2 within the resolution of data. The spectral decorations indicated a predominantly tetragonal crystalline structure with a minimal amount of monoclinic crystalline structure in all groups, as presented in Figure 2D. At a Bragg angle of 30.177°, the t- peak intensities strongly indicated that it correlated with the t-crystalline spectra from the XRD standard file of ZrO2. The low peaks intensity of the t-spectra also exhibited at 2θ of 35.172° and 34.607°, which corresponded with the standard crystal spectra at t-phase. The detectable peaks at 2θ of 31.119°and 27.792° correspond with the crystal spectra in m-phase, as designated from the XRD file of ZrO2. The relative concentration (wt.%) of the m-phase regarding the total zirconia phase revealed the variation in the amount of phase metamorphosis from t→m-phase as a result of the differences in varied sintering temperatures and lengthening sintering duration, as presented in  Table 1. The diversification of phase concentration (t- and m- phase) in weight percentage were 0.6972, 0.3028 for SIHP, 0.7180, 0.2820 for SIHR, 0.7258, 0.2742 for SRHP, 0.7476, 0.2524 for SRHP, 0.7736, 0.2264 for SRHR, 0.8084, 0.1916 for SRHS, 0.8222, 0.1778 for SDHP, 0.8461, 0.1539 for SDHR, and 0.8462, 0.1538 for SDHS. The relative composition of the phase considerably differed and correlated with the sintered process of Y-TZP. The composition for m-spectra elevated upon the sintered Y-TZP at greater sintered temperature along with the extended sintering time. It suggested that the phase conversion phenomenon (t- → m- phase) was established upon raising the sintered temperature together with the lengthening sintering time, as presented in  Table 1 and Figure 2D.

**Figure 3:**
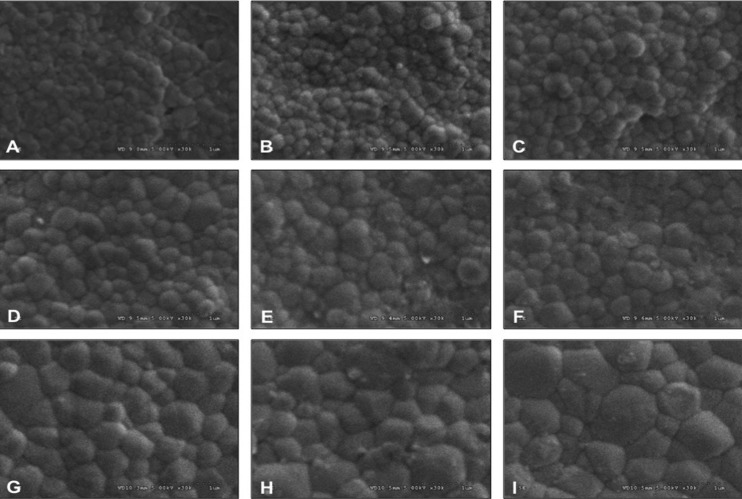
SEM photomicrographs indicated grain size and grain distribution of Ceramill Zolid® monolithic zirconia, sintered at decreasing- (A, B, C), regular- (D, E, F), and increasing- (G, H, I) sintered temperature, with shortening- (A, D, G), regular- (B, E, H), and prolonged- (C, F, I) sintering time at X30K magnification.

The SEM photomicrograph was used to evaluate the size of grain arrangement for monolithic YTZP. The grain size for each group revealed differences as a result of the different sintering processes, as indicated in Figure 3 and  Table 1. Upon sintering monolithic Y-TZP at reducing sintered temperatures, the SEM photomicrograph predominantly exhibited crystalline microstructures in fine grain (ranging from 0.1–0.4 μm). Increasing the sintered temperature contributed to an enlargement of the grain size, as indicated by an increase in the amount of medium grain size (ranging from 0.5–0.8 μm) and large grain size (ranging from 0.9–1.3 μm). Relative percentages of the grain size (fine, medium, and large) presented with 100, 0, 0 for SDHS, 98.19, 1.81, 0 for SDHR, 98.16, 1.84, 0 for SDHP, 97.26, 2.74, 0 for SRHS, 87.5, 12.5, 0 for SRHR, 65.39, 34.61, 0 for SRHP, 47.69, 49.23, 3.08 for SIHS, 14.28, 63.28, 22.44 for SIHR and 10.45, 64.17, 25.78 for SIHP, accordingly. The sintered process of monolithic zirconia aimed at raising the sintered temperature demonstrated crystalline microstructure in medium sizes to a greater extent than sintering at either regular- or reducing-sintered temperatures. The result also indicated that the longer the extending sintering time, the greater the amount of grain enlargement exhibited, as shown in  Table 1 and Figure 3. Nevertheless, such extended sintering time seemed to affect the enlargement of grain of Y-TZP less, when compared to raising the sintered temperature. The micrographs also exhibited faulty assimilation of crystal structure at grain boundaries for reducing the sintered temperature and shortening the sintering time in groups. On the other hand, the crystal structures demonstrated densely packed crystalline microstructures in groups of raising sintered temperature and extended sintered time.

## Discussion

The investigation endeavored to determine the feasibility of obtaining superior color improvement in high translucency monolithic Y-TZP upon alteration of the sintering process. The altered sintered parameter is careful about practicing dentistry since the query that dealt even with the optical properties of the monolithic zirconia shall be obtained to meet a natural looking tooth appearance and enable better translucency by varying the sintering time and sintered temperature. The probability of the phase mutation (t- → m-phase) might be uncertain while changing the sintered procedure and might contribute to the variation of the optical parameters of zirconia. The possible advantage was that a suitable sintered process without endangering optical characteristics of the Y-TZP restoration might be profitable for constructing zirconia dental prosthesis available for insertion quicker. The research determined optical properties of the monolithic zirconia by measuring the spectral reflectance and calculated it for TP as in other studies (11,17,24). The scrutiny revealed that altering the sintering time and sintered temperature affected the translucency of zirconia. This stipulated that altering the sintering process significantly influenced the translucency of zirconia. Hence, the null hypothesis was not accepted.

**Figure 4:**
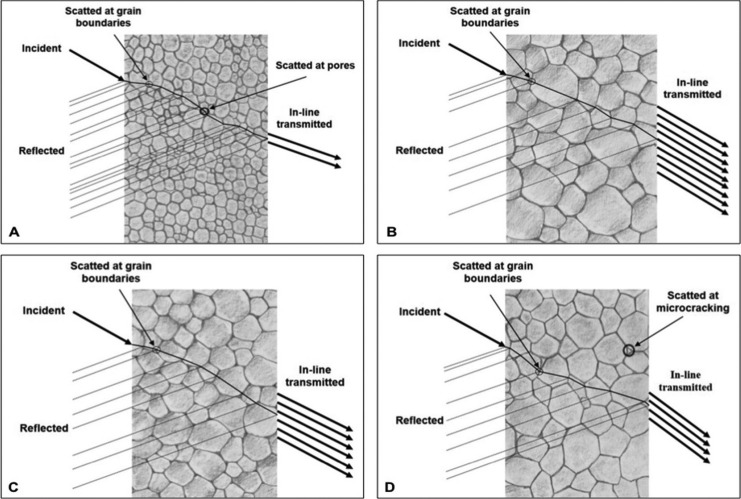
Possible explanation the behavior of light in reflection, scattering, and transmission in relation with grain sized, grain boundary, and pores (A), with increasing spectral translucence upon extending sintering time (B), upraising sintered temperature (C), while nano-crack in grain boundary exhibited as sintering at extremely high temperature together with long duration of sintering time (D).

For the prospect of translucency, it is a paramount that the color characteristics for simulating the color appearance of tooth is exemplified as crucial determinants in the deliberation for material selection in prosthodontic reconstruction, specifically in the visible region (8). The translucence property of zirconia was significantly increasing upon increasing the sintered temperature and prolonging the sintering time. The monolithic zirconia seemed to possess minimal difference in translucency regardless of the sintered performance at regular- versus raising-sintered temperature together with the sintering time of regular- versus prolonged-sintering time. Contrarily, the optical translucence of zirconia dreadfully diminished as the zirconia was sintered at a reducing sintered temperature or decreasing sintering time. This phenomenon possibly correlated with the completion of the crystallization of Y-TZP in addition to the depreciation in confined defects at the grain boundaries as well as the grain growth phenomenon. The grain size enlargement was perceived in case of either upraising the sintered temperature or lengthening the sintered-holding time, which was presumably capable of slackening the micro-pores and their allocation at the border of the grain of poly-crystalline structure by expediting the dispersion ability among atoms using an atomic attraction force. Regarding upraising the sintered temperature or lengthening sintering time, the particles of Y-TZP were able to fuse together, producing shrinkage of the pore size between the borders of grain during the solidified diffusion stage and enhancing the density of zirconia. The situation was well described by the XRD pattern, illustrating that the crystal structure shift from t- → m-phase in conjunction with the SEM photomicrograph expressed the growth of nano-sized grains of zirconia against upraising the sintered temperature and lengthening the sintering time. Accordingly, the combinative effect of the porous shrinkage and compactness of nano-crystalline zirconia was presumably increasing in the homogeneity of crystal structure, which ultimately encouraged finer specular reflectance and optical transmittance with minimized refraction, as illustrated in Figures 4 (A) and (B). This is apparently the principal reasoning of this study to point out that upraising the sintered temperature to an optimal level can obtain better translucency than reducing the sintered temperature together with lengthening the sintering process convinces more translucence than either shortening- or supporting regular-sintering duration, which was supported by other studies (14,17,21,22,24).

Nonetheless, better achieving more translucency of sintered nano-sized zirconia somewhat declined as the sintered temperature extended to 1550°C; this is possibly associated with the utmost increase of the m-phase accompanying the origination of a micro-crack in the m-phase itself, which possibly acts as a teeny defect in the crystalline microstructure as described in other studies (11,15). These teeny cracks may inaugurate scattering effects as well as debilitate diffusion translucency and lead to an interruption in the agreeable translucency, as illustrated in Figures 4 (C) and (D). This study advised that varying the sintering procedure significantly influenced the translucency of nano-sized monolithic zirconia. It apparently proved that bettering the translucency of nano-sized monolithic zirconia is feasible by means of upraising the sintered temperature or lengthening the sintering time. Contrarily, lowering the sintered temperature or minimizing sintering time may imperil the translucency of nano-sized zirconia.

## Conclusions

This study indicated that translucency of nano-crystal zirconia was influenced by altering sintered procedure. Significant improvements in translucency was capable through upraising the sintered temperature and prolonging the sintering time, which provided significant meaning about the optical property of zirconia for providing esthetic achievement of restoration. Reducing the sintered temperature and shortening the sintering time can compromise translucency; however, it still benefits the expediting restoration fabrication that probably considered the imperceptible area. Ultimately, the color appearance improvement in the nano-crystalline monolithic zirconia is feasible by alternating the sintering process via upraising sintered temperature or prolonging sintering time and is advised for accomplishing the sintering agenda.

## Clinical significance

Improving spectral translucency of nano-sized Y-TZP is feasible through varying sintering procedure. Sintering process of Y-TZP at high sintered temperature and extended holding time is capable of rendering more translucence of zirconia restoration and is recommended for sintering procedure to achieve for esthetic zirconia restoration.
